# Highly Efficient Color Tuning of Lithium Niobate Nanostructures on Flexible Substrate

**DOI:** 10.3390/ma18051006

**Published:** 2025-02-25

**Authors:** Weiming Zhang, Shifeng Dai, Fengji Wu, Shifa Pan, Jianzhi Su, Pinghui Wu, Lina Cui

**Affiliations:** 1 Fujian Provincial Key Laboratory for Advanced Micro-Nano Photonics Technology and Devices & Key Laboratory of Information Functional Material for Fujian Higher Education, Quanzhou Normal University, Quanzhou 362000, China; zwm08540854@163.com (W.Z.); dsf0114@163.com (S.D.); 15671200023@163.com (F.W.); panshifa@qztc.edu.cn (S.P.); sujianzhi@qztc.edu.cn (J.S.); 2 College of Textiles and Apparel, Quanzhou Normal University, Quanzhou 362000, China

**Keywords:** lithium niobate, polydimethylsiloxane, nanostructure, structural color, reflection

## Abstract

Nanostructures based on flexible material are essential for modulating reflected colors by actively changing the unit structure. However, current nanostructures face challenges in achieving active and efficient modulation across a broader spectral range. Here, we propose a stretchable color management method. The structure consists of a polydimethylsiloxane (PDMS) flexible substrate and cross-shaped lithium niobate (LiNbO_3_). This study achieves reflection color changes, continuous adjustment, and automatic switching of solar spectrum reflectance by optimizing the geometric structure. It shows that the spectral tuning range is larger, benefiting from the special nanostructures and the stretchability of PDMS, which result in a larger tunable period range and a maximum wavelength shift of nearly 180 nm. Moreover, this unique design has been effectively balanced and optimized to respond to different polarization waves. Finally, the sensing characteristics of the nanostructure are studied through its response to changes in the refractive index (RI). The results demonstrate a method with implications for flexible electronic devices, color generation, and biochemical sensing, contributing to progress in flexible wearable technology and green building.

## 1. Introduction

In the last few years, flexible optical devices have evolved into a dynamic and rapidly advancing field. This development is driven by continuous innovation in materials and methods to meet the diverse needs of applications [1,2,3,4]. One of the key challenges in this field is the ability to actively and efficiently adjust the reflected color, which has been widely recognized as a core element. In this context, the integration of nanostructures on stretchable substrates is seen as an approach to address these challenges, providing a viable platform for dynamic color variation and reflection control [5,6]. The polydimethylsiloxane (PDMS) is known for its exceptional optical transparency, biocompatibility, processability, thermal stability, and chemical resistance [7,8,9,10]. Utilizing the stretchable properties of PDMS substrates [11,12], the proposed structure achieves color changes by reflecting and actively tuning the periodicity of the unit cell through mechanical adjustment. PDMS has been considered the material of choice for many emerging technologies, including flexible electronic devices [13,14,15], biomedical equipment [16,17,18,19], and structured surfaces [20,21,22]. Lithium niobate (LiNbO_3_), a composite crystal primarily composed of lithium, niobium, and oxygen elements, has sparked widespread study interest in the scientific community. It holds a unique position in the field of optoelectronics, primarily due to its exceptional physical and electro-optical properties, as well as its relative ease of preparation. Lithium niobate has a series of excellent properties, such as a high refractive index, excellent electro-optical effects, and stability, which are important for various advanced technologies for controlling or manipulating light [23,24,25]. LiNbO_3_ and PDMS play crucial roles in constructing efficient and reliable flexible optical devices.

In the context of this technology, this study aims to achieve efficient color tuning of lithium niobate nanostructures on stretchable substrates, advancing the latest developments in flexible optical device technology to meet the growing demands of various applications. Specifically, LiNbO3 micro-nanostructures on PDMS substrates can efficiently tune reflective colors through mechanical manipulation of unit structures [26,27]. It has different functions in different fields. In the field of flexible electronics, Zhang et al. proposed a highly stretchable and flexible hydrogel film with structural color. The film uses natural and synthetic polymers (PAM, SF, PEDOT: PSS, and GO) to create electronic skin. It can change color and respond to electricity in real time when people move [28]. In color generation, Zhang et al. suggested using a simple co-assembly method. The method creates printable circularly polarized luminescence (CPL) photonic paints. This allows the creation of structural colors in flexible coatings that can be meters long and applied to different surfaces. Such innovation helps develop flexible 3D display panels and future wearable electronics with better structural color and flexibility [29]. In the field of biochemical sensing, Hu et al. proposed a structural color hydrogel to detect exhaled volatile sulfur compounds (VSCs) by the naked eye. It has a detection range of 0–1 ppm and a 61 ppb limit. This allows for visual monitoring of *Porphyromonas gingivalis*. By integrating this hydrogel into a sensor array with smartphone color analysis, they offer a portable point-of-care testing (POCT) solution for halitosis monitoring and periodontitis screening [30]. These applications support flexible wearable technology. They also provide new ideas for green building innovation [31]; for example, Sun et al. proposed self-adaptive photochromism (SAP) materials using donor–acceptor Stenhouse adducts (DASAs) and organic dyes. These materials change color in response to light, offering an innovative green building technology for active camouflage. It adapts to backgrounds, reducing complexity and cost [32]. This innovative approach represents progress in color variation capabilities. It also opens new avenues for developing flexible optical devices with better functionality and performance. For deeper insight, the interaction between LiNbO_3_ nanostructures and flexible substrates can better understand the mechanism of color modulation and further optimize its performance [33].

The integration of LiNbO_3_ nanostructures with a flexible substrate offers a promising avenue for exploration. Song et al. proposed a metal nanoparticle and PDMS composite structure, which can relatively easily achieve dynamic spectral tuning. By stretching, the resonance-induced color can be dynamically tuned from green to purple [34]. Zhou et al. proposed a nano-disc structure made of LiNbO_3_, achieving over 80% high reflectance efficiency. Furthermore, they demonstrated the output colors of different array units composed of individual nano-discs, laying a theoretical foundation for practical applications [35]. Zheng et al. presented a metamaterial consisting of visible light LiNbO_3_ nano-ring (LNR) structures on a PDMS substrate. This metamaterial utilizes mechanical tuning of the periodicity of unit structures to actively adjust the reflective colors [36]. Recently, Xu et al. introduced a structure where LiNbO_3_ nano-dimers are arranged on a PDMS substrate, achieving active tuning of reflective colors through mechanical tuning [37]. Compared with this study, its reflection efficiency is not high and the range of color change is not large. Either its periodic tuning range is large but the spectral shift distance is short or its periodic tuning range is small but the spectral shift distance is slightly larger. In this study, not only can the tunable period range reach nearly 230 nm but, also, the maximum spectral shift is close to 180 nm, enabling efficient color tuning. A significant challenge is achieving high chromaticity for active reflection tuning while ensuring efficient color adjustment. These aspects have been identified as key areas requiring enhancement for optimal performance. Viewing these challenges as opportunities for innovation, we introduce a cross-shaped LiNbO_3_ structure based on a PDMS substrate as part of a stretchable color management method.

The core of the proposed methodology lies in the fine optimization of geometric configurations, which is the key step to enhancing the active tuning capability of reflective colors. By utilizing the unique optical properties of the cross-shaped structure and combining the stretchability of the PDMS substrate, a significant expansion of the spectral tuning range has been demonstrated. This achievement surpasses traditional nanostructures. It marks a significant breakthrough in color variation and optical control. In addition, the maximum spectral tuning range achieved is close to 180 nanometers, which is not only impressive but also marks an advancement in dynamic color control. By precisely controlling nanostructure geometry and leveraging flexible substrates, it achieved efficient color tuning, laying a solid foundation for next-generation optical technology. This breakthrough supports flexible wearable technology and stretchable electronics and opens new pathways for optical technology development. Looking forward, this achievement is expected to drive ongoing innovation in flexible optical devices, supporting the advancement of next-generation optical technologies with higher flexibility and performance.

## 2. Structure and Methods

**Figure 1:**
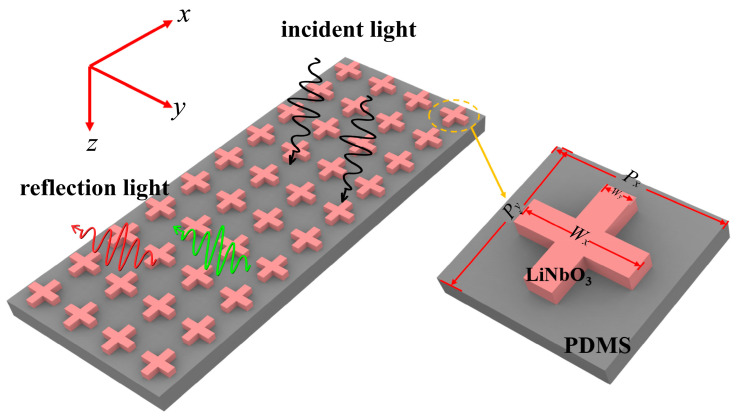
Schematic representation of cross-shaped LiNbO_3_ on a PDMS substrate in an academic context.

Figure 1 shows a detailed illustration of the schematic structure of a cross-shaped LiNbO_3_ on a PDMS substrate. This metasurface structure consists of LiNbO_3_ cuboids intersecting in a cross shape, firmly positioned on the stretchable PDMS substrate. This design allows precise spectral tuning through mechanical means, enabling active tuning of reflection colors. Figure 1 shows the detailed geometry of the proposed unit structure, including specific geometric parameters of the two intersecting cuboids forming the cross shape. This study is based on FDTD simulation, and a preparation method will be introduced here. Cross-shaped LiNbO_3_ nanostructures were prepared by photolithography [38] and reactive ion etching [39], then spin-coated with PDMS and solidified to form a substrate. The nanostructure morphology was verified by scanning electron microscopy (SEM), and the reflectance spectrum was measured by an ultraviolet–visible–near-infrared spectrophotometer (PerkinElmer, Shelton, CT, USA, Lambda 1050). In this structure, the lattice constants *P_x_* and *P_y_* represent the periodicity along the structure’s *x*- and *y*-axis directions, with the height *h* of the LiNbO_3_ cuboids. Additionally, *W_x_* and *W_y_* denote the length and width of the LiNbO_3_ cuboids. Through this carefully designed unit structure, it aims to achieve a broader spectral tuning range, providing strong support for enhancing performance and expanding the applications of flexible optical devices.

In this study, it is firmly believed that the Finite-Difference Time-Domain (FDTD) method based on the vector electromagnetic wave theory can accurately describe the propagation of light waves in media. To simulate the structure of the cross-shaped LiNbO_3_ metasurface on a PDMS substrate, using smaller grid sizes can improve the accuracy of simulation results, but it also needs much more computing power and time. This study uses a nonuniform grid FDTD method to keep the results accurate and reliable. The smallest grid size is set at 3 nm. This helps to correctly show how light waves move and also keeps the simulation fast; we balance between obtaining precise results and not taking too long to run the simulations. As a flexible substrate, the thickness of PDMS directly affects the stretchability and mechanical tuning ability of nanostructures. Thicker PDMS offers more flexibility and strength [40], and it keeps the nanostructures in better shape. So, we chose a PDMS thickness of 400 nm for this study. This thickness makes sure the material is flexible enough for mechanical tuning and also works well optically. During the simulation, perfectly matched layers (PML) were placed around the boundaries. This absorbed the reflected waves and prevented them from affecting the results. Considering the periodicity of the metasurfaces, it is only necessary to calculate a single-unit structure. Then, to simulate the optical response of the entire metasurface by applying periodic boundary conditions in the *x* and *y* directions. Additionally, perfectly matched layer boundary conditions were set up along the *z* direction perpendicular to the surface to ensure efficient absorption of light waves in the *z* direction. To calculate the spectral tuning range, power monitors were placed on the *x–y* plane to record changes in the reflected spectra. In the simulation process, the electric field € and magnetic field (*H*) were oriented along the *x* and *y* directions, respectively, while the wave vector (*k*) was directed along the *z* direction, by the propagation laws of electromagnetic waves, accurately reflecting the optical properties of the metasurface. Through this series of simulation settings and calculation processes, this study aims to delve into the optical performance of the cross-shaped lithium niobate metasurface, providing strong theoretical support for the design and optimization of flexible optical devices.

## 3. Numerical Results and Discussion

**Figure 2:**
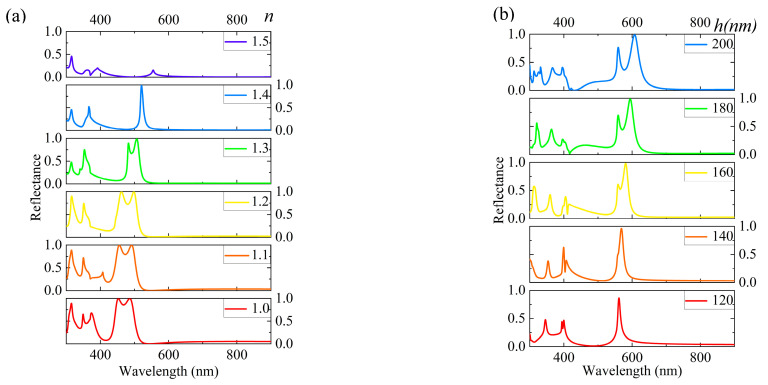

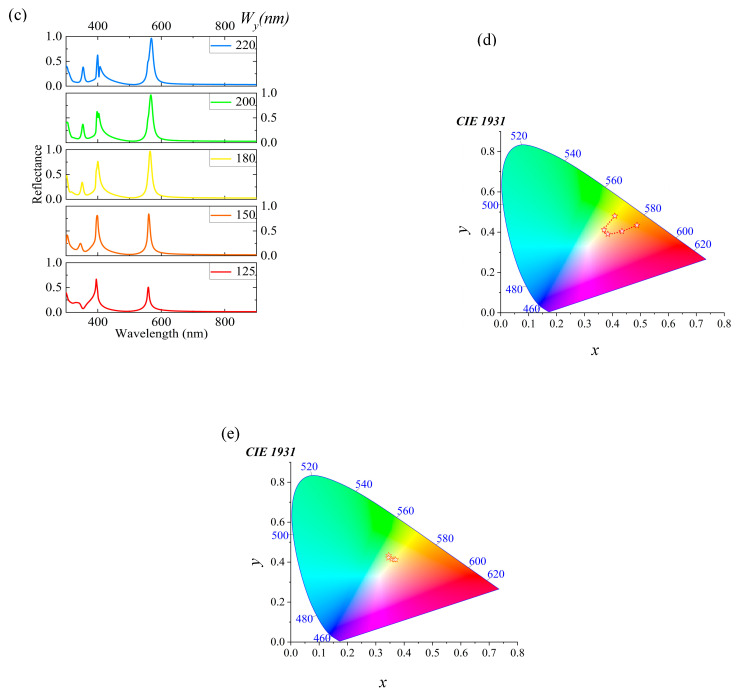
The optical properties of micro-nanostructure are influenced by substrate and structural parameters analysis. (**a**) The reflection spectrum of micro-nanostructure as a function of substrate refractive index. (**b**) The effect of structural height variation on the resonance peak position. (**c**) The impact of structural width variation on the resonance peak and reflectance. (**d**,**e**) The spectral trends at different heights and widths through trend lines.

Firstly, we deeply explored the influence of the substrate on the simulation results to gain a more accurate understanding of the optical properties of nanostructures. Figure 2a shows the variation in the substrate’s refractive index (*n*) from 1.0 to 1.5 to observe its effect on the reflection spectrum. For example, the refractive index of silica (SiO_2_) is approximately 1.46 and that of magnesium fluoride (MgF_2_) is around 1.38. The experimental results indicate that, with an increase in the refractive index, multiple reflection peaks gradually decrease and eventually transform into a single resonance peak until they completely vanish. This phenomenon provides us with an effective means to manipulate the spectrum, further confirming the crucial role of the substrate properties in the optical behavior of nanostructures. The structural parameters of nanostructures have an impact on their optical properties, which is another important pattern discovered during the study. To elaborate on this pattern, we took a set of default configuration parameters as an example, namely *P_x_ = P_y_* = 400 nm, *h* = 140 nm, *W_x_* = 250 nm, and *W_y_* = 220 nm. By altering these parameters, the spectrum can be finely controlled. As shown in Figure 2b, when the *h* value gradually increased from 120 nm to 200 nm, the resonance peak red shifts from 561.7 nm to 607.4 nm, covering a range of 45.7 nm. It is noteworthy that, within the range of *h* ≥ 120 nm, the reflection efficiency remains above 91%, demonstrating excellent optical performance. Specifically, reducing *h* to 140 nm suppresses multiple reflection peaks in Figure 2b, the color change from yellow to orange, as evidenced by the red trend line in Figure 2d. Furthermore, we investigated the influence of width (*W_y_*) on the spectrum. As shown in Figure 2c, when *W_y_* changes from 125 nm to 220 nm, the resonance peak red shifts from 559.4 nm to 568.9 nm, a small but noticeable spectral change. Particularly, for *W_y_* ≥ 180 nm, the reflection efficiency reaches above 99%, showcasing the excellent reflection performance of the structure under specific parameters. Interestingly, as *W_y_* increases, the generated colors become purer yellow, as validated by the orange trend line in Figure 2e. This finding provides us with an effective method to achieve color purification by adjusting the structural parameters. This study shows how the substrate and structural parameters affect the optical properties of nanostructures. It also offers an effective way to control the spectrum by fine-tuning parameters.

**Figure 3:**
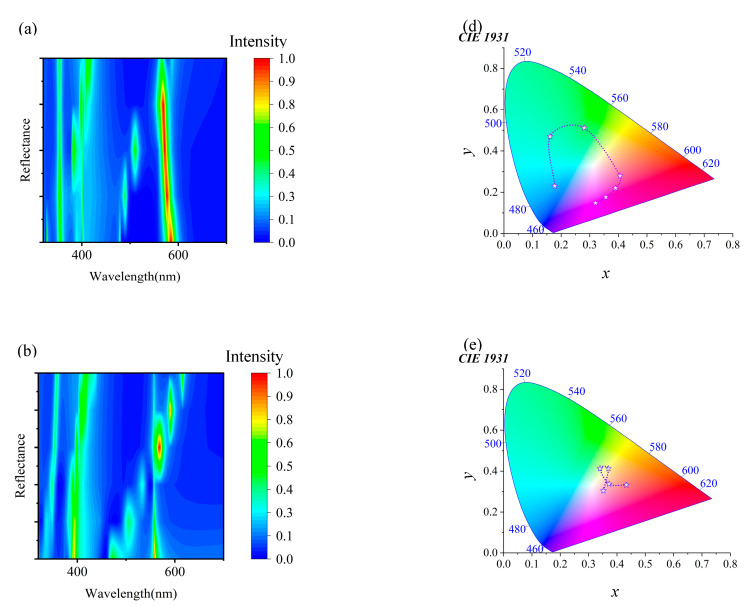

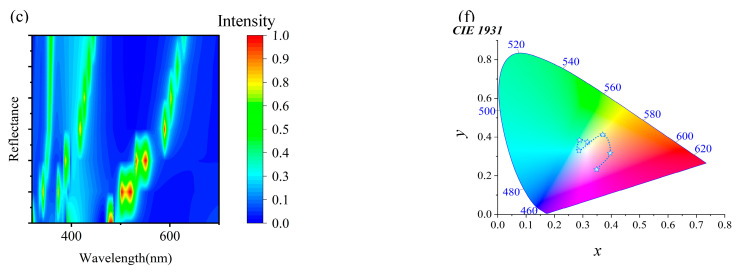
The impact of the micro-nanostructure period on the spectrum and color is analyzed. (**a**) Detailed description of the movement of the reflection peak in the reflection spectrum as the period *P* increases gradually from 280 nm to 450 nm. (**b**) The changes in the reflection spectrum by keeping *Px* constant and only varying *Py*. (**c**) The changes in the reflection spectrum by keeping *Py* constant and changing *Px*. (**d**–**f**) The color trends under different conditions through trend lines.

Changing the periodicity of micro-nanostructures affects the reflection spectrum. This gives important proof to understand their optical properties. See Figure 3. Initially, as the period (*P*) gradually increases from 280 nm to 450 nm (denoted as *P = P_x_ = P_y_*), it can be observed in Figure 3a that the reflection peak red shifts from 479.6 nm to 630.2 nm, covering a range of 150.6 nm. This phenomenon typically occurs in periodic structures, where changes in periodicity lead to alterations in the diffraction conditions, thereby affecting the wavelength of the reflected light. Specifically, as the period increases, the wavelength that satisfies the Bragg condition also increases, resulting in an observed shift in the reflection peak towards longer wavelengths (red shift). Throughout this process, wavelengths with high reflectivity exhibit a pronounced red shift as *P* increases from 280 nm to 440 nm. However, when *P* continues to increase to 450 nm, the reflection peak rapidly disappears, indicating a significant change in the reflection performance of light by the structure at a specific period. This change is visually represented by rich color variations spanning the blue–green–red regions, as shown in Figure 3d. Subsequently, we investigated the variation in the reflection spectrum when keeping *P_x_* constant and altering *P_y_*. In Figure 3b, maintaining *P_x_* at 400 nm while increasing *P_y_* from 280 nm to 420 nm leads to a blue shift of the reflection peak from 566.5 nm to 583.7 nm, covering a range of 17.2 nm. Notably, as *P_y_* increases from 280 nm to 400 nm, although wavelengths with high reflectivity experience a blue shift, the change is not significant. This phenomenon may be attributed to more electric field energy being concentrated together, forming a vortex flow along the *x*-axis, thereby affecting the characteristics of the reflection spectrum. This change in color transitions from the red region to the green region, eventually progressing towards purer colors, as depicted in Figure 3e. Finally, the variation in the reflection spectrum was examined when keeping *P_y_* constant and changing *P_x_*. In Figure 3c, maintaining *P_y_* at 400 nm while increasing *P_x_* from 280 nm to 440 nm, it is observed that, as *P_x_* increases from 280 nm to 360 nm, the resonance peak gradually diminishes. However, when *P_x_* reaches 400 nm, the reflection peak rapidly appears with high reflectivity, exceeding 99%. In terms of color, this variation manifests as a shift from pure colors towards the green region. When the *P_x_* period reaches 360 nm, the color rapidly becomes pure and, as the *P_x_* period increases, the color eventually shifts toward the red region. This outcome suggests that, by adjusting the value of *P_x_*, precise control over the reflection spectrum can be achieved, enabling effective color control. By altering the periodicity of the micro-nanostructure, the reflection spectrum is finely tuned, thereby achieving rich color variations. This study helps understand the optical properties of micro-nanostructures. It also provides new technical support and solutions for developing optical filters, display technologies, anticounterfeiting labels, and more.

**Figure 4:**
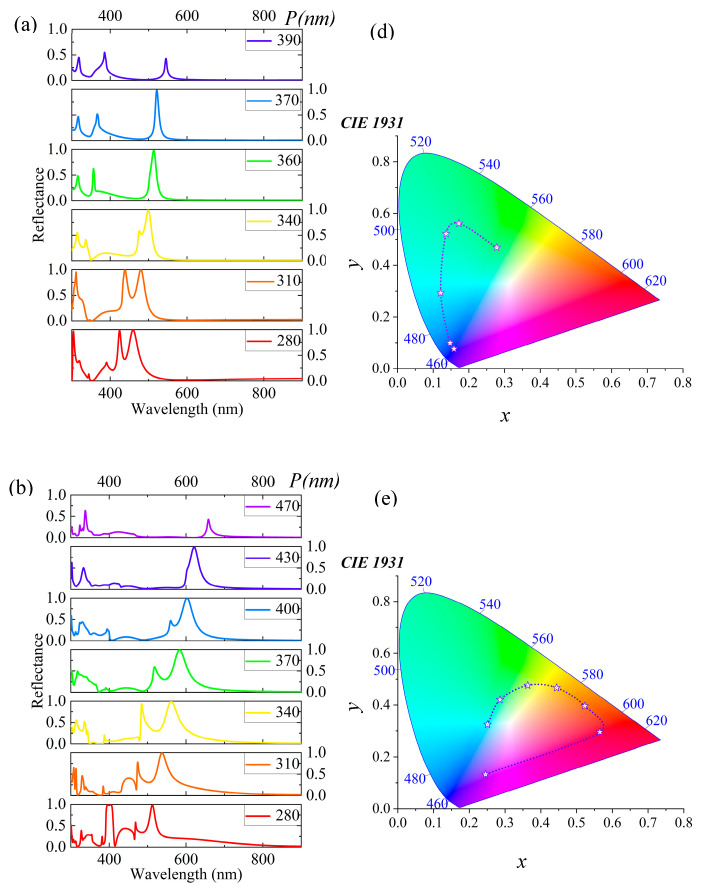

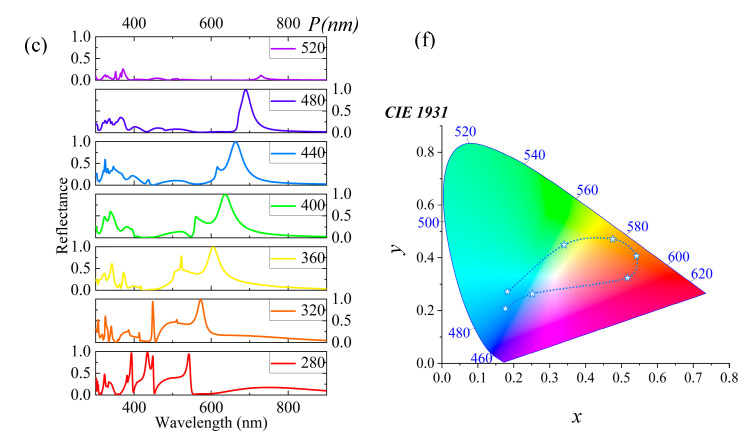
Control of reflection spectra and colors through mechanical stretching of micro-nanostructures on a PDMS substrate. (**a**) The spectral changes of a micro-nanostructure with specific parameters (*W_x_* = 200 nm, *W_y_* = 170 nm, *h* = 150 nm) as period *P* increases from 280 nm to 390 nm. (**b**) The spectral response of another structure (*W_x_* = 220 nm, *W_y_* = 190 nm, *h* = 250 nm) as the period varies. (**c**) The spectral changes of a micro-nanostructure with size parameters (*W_x_* = 240 nm, *W_y_* = 210 nm, *h* = 300 nm) as the period changes. (**d**–**f**) The color trends under different conditions through trend lines.

Due to the inherent stretchability of PDMS as a flexible substrate material, it provides the possibility for mechanical tuning of micro-nanostructures. Applying strain to PDMS will alter the periodic arrangement of the micro-nanostructures through mechanical action, thereby achieving precise control over the spectrum. Figure 4a–c show the reflection spectrum changes for three different geometric configurations during mechanical stretching. The specific parameters of these structures are detailed in Table 1, showcasing varying spectral response characteristics by adjusting parameters such as width (*W_x_* and *W_y_*) and height (*h*).

The micro-nanostructure parameters are *W_x_* = 200 nm, *W_y_* = 170 nm, and *h* = 150 nm in Figure 4a. As period *P* increases from 280 nm to 390 nm, the reflection peak significantly red shifts from 460 nm to 545.8 nm, covering a range of 85.8 nm. Meanwhile, the full-width half maximum (FWHM) of the reflection peak gradually narrows until the peak disappears. These spectral changes show a continuous color transformation from deep blue to green to yellow–green in the visible light range. The purple trend line in Figure 4d visually demonstrates this color change trend. In Figure 4b, another structure is shown with parameters *W_x_* = 220 nm, *W_y_* = 190 nm, and *h* = 250 nm. As period *P* increases from 280 nm to 470 nm, the reflection peak red shifts from 512.5 nm to 657.9 nm, covering a range of 145.4 nm. This change extends the reflection spectrum from deep purple to the red region, eventually entering the green region, spanning three different color domains. By stretching the PDMS substrate to alter the period, efficient color tuning is achieved, as indicated by the deep purple trend line in Figure 4e. Figure 4c illustrates a micro-nanostructure with parameters *W_x_* = 240 nm, *W_y_* = 210 nm, and *h* = 300 nm. As period *P* increases from 280 nm to 500 nm, the reflection peak red shifts from 539.8 nm to 719.6 nm, covering a range of 179.8 nm. During this process, multiple reflection peaks gradually transform into a single peak, demonstrating the flexibility and efficiency of spectral control by the structure. The red shift in the reflection wavelength results in a continuous color change in the visible light range, transitioning from light blue, tender green, orange, dark orange, magenta, and purple to blue, as shown by the light blue trend line in Figure 4f. These phenomena above can be explained by Bragg diffraction conditions and resonance effects. As the period increases, the wavelength that satisfies the Bragg condition also increases, leading to a red shift of the reflection peak. Additionally, as the structure’s internal resonance conditions change with the period, different wavelengths of light are more effectively reflected or transmitted.

**Figure 5:**
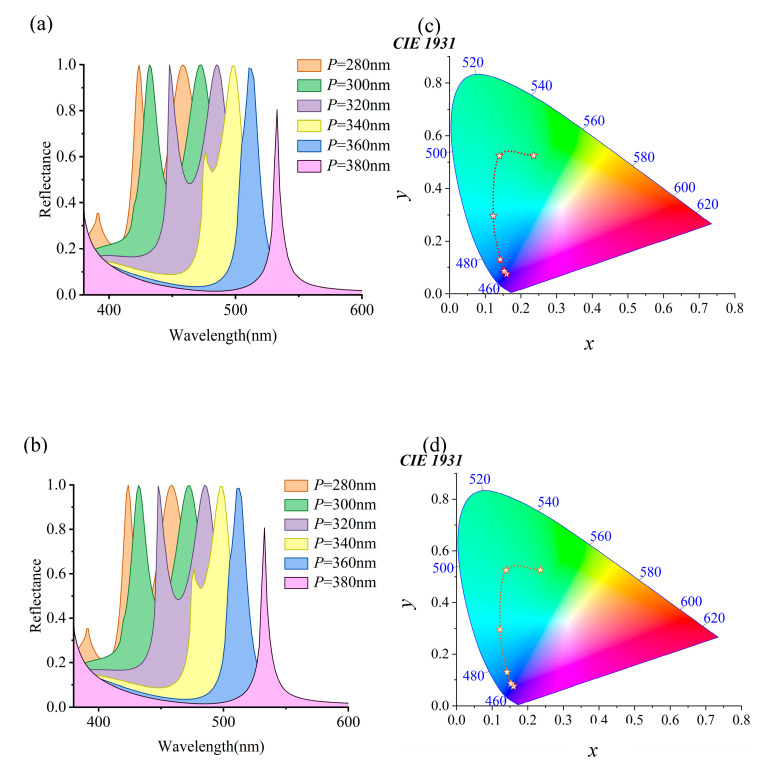

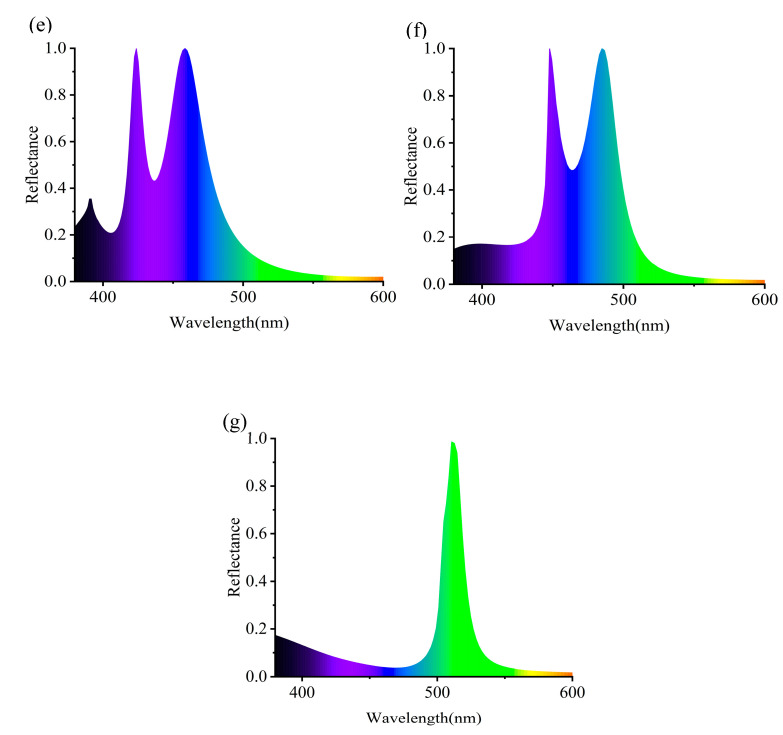
Investigation into the influence of polarization states on metasurface optical properties. (**a**) The spectral distribution under TE mode, detailing specific spectral features. (**c**) The color change pattern corresponding to the reflection spectra under TE mode. (**b**) The spectral distribution under TM mode. (**d**) The color changes corresponding to the reflection spectra under TM mode. The changes in reflectance spectra of the structural surface under different periodic conditions (**e**–**g**), along with the corresponding trends in color variation; the period is 280 nm, 320 nm, and 360 nm.

Subsequently, we investigated the specific manifestations of polarization states. Figure 5 shows that the spectra of transverse electric (TE) and transverse magnetic (TM) modes almost completely overlap. This phenomenon shows that the structure responds similarly to different polarization waves. In other words, within the studied frequency range, polarization states have minimal effect on the metasurface’s optical properties and can be ignored. Within the simulated frequency range, TE and TM polarization waves show similar propagation and reflection, resulting in nearly identical spectra. This discovery suggests that the metasurface structure designed has effectively balanced and optimized the response to different polarization waves, eliminating the need for specific control tailored to individual polarizations. This study not only enhances the understanding of polarization states but also provides a new perspective and approach for metasurface design, potentially offering impetus to the development of spectral control technologies. To further clarify this trend in color variation, Figure 5e–g illustrates the changes in reflection spectra as the periodicity varies, highlighting the corresponding shifts in structural color. It is evident that, when the reflection reaches its peak, it aligns with the observed color transitions on the 1931 CIE color space diagram.

**Figure 6:**
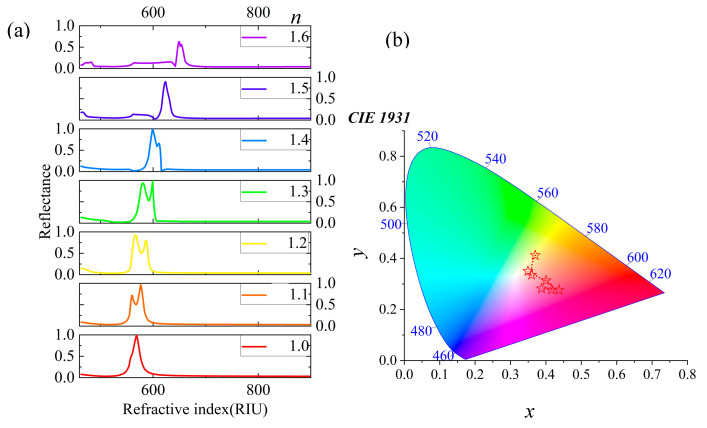
(**a**) The change of reflection spectrum when the environmental RI *n* increases from 1.0 to 1.6. (**b**) The CIE model of the reflectance spectra.

Then, the sensing performance of the nanostructure is studied as shown in Figure 6. Figure 6a shows the change in the reflection spectrum of the nanostructure in the process of changing the RI *n* from 1.0 to 1.6. The peak wavelength of the nanostructure will be red shift and the reflection efficiency is almost unchanged; only when *n* = 1.6 does the reflection efficiency decrease. Here, this phenomenon is attributed to the fact that the increase in sensing medium value can increase the damping of the dipole mode. The peak wavelength of the reflection spectrum has shifted from 568.9 nm to 648.4 nm, covering a range of 79.5 nm. Figure 6b shows that the reflection spectrum is highlighted by the CIE model, with the color changing from yellow through red to purple.

## 4. Conclusions

This study highlights the significance of efficiently tuning the reflection color variation of LiNbO_3_ nanostructures on a PDMS substrate. It also proposes a novel cross-shaped LiNbO_3_ structure for stretchable color management systems. The system not only enables flexible changes in reflection color with nearly 100% high reflection intensity. It also achieves continuous adjustment of solar spectrum reflectance and automatic switching functions. By optimizing the geometric structure, the ability to actively tune reflection colors is enhanced, demonstrating a larger tunable period range compared to other micro-nanostructures, with a maximum range close to 180 nm. This improvement is largely attributed to the outstanding stretchability of the PDMS material and unique nanostructures. This achievement provides new methods and technological support for flexible electronic devices, color generation, and biochemical sensing, potentially driving rapid advancements in these fields. Particularly in practical applications, such as flexible wearable technology and green buildings, the proposed method holds potential value and broad application prospects. In summary, efficient tuning of reflection colors and continuous adjustment of spectral reflectance is achieved by designing a novel cross-shaped LiNbO_3_ structure, demonstrating important application value across multiple fields. In the future, efforts will continue to explore performance optimization and practical applications of this structure to make greater contributions to the development of relevant fields.

## Figures and Tables

**Table 1:** Feature sizes and optical performances for various colors.

*W_x_* (nm)	*W_y_* (nm)	*H* (nm)	Spectral Shift Range (nm)
200	120	150	85.8
220	190	250	145.4
240	210	300	179.8

## Data Availability

The original contributions presented in this study are included in the article. Further inquiries can be directed to the corresponding authors.
